# Large miRNA survival analysis reveals a prognostic four-biomarker signature for triple negative breast cancer

**DOI:** 10.1590/1678-4685-GMB-2018-0269

**Published:** 2020-03-02

**Authors:** Fernando Andrade, Asuka Nakata, Noriko Gotoh, André Fujita

**Affiliations:** ^1^ Universidade de São Paulo, Programa Internunidades de Pós-Graduação em Bioinformática, São Paulo, SP, Brazil.; ^2^ Kanazawa University, Cancer Research Institute, Division of Cancer Cell Biology, Kanazawa, Ishikawa, Japan.; ^3^ Universidade de São Paulo, Faculdade de Medicina, Departamento de Pediatria, São Paulo, SP, Brazil.; ^4^ Universidade de São Paulo, Instituto de Matemática e Estatística, Departamento de Ciência de Computação, São Paulo, SP, Brazil.

**Keywords:** miRNA, miR-221, miR-1305, miR-4708, RMDN2

## Abstract

Triple negative breast cancer (TNBC) is currently the only major breast tumor subtype without effective targeted therapy and, as a consequence, usually presents a poor outcome. Due to its more aggressive phenotype, there is an urgent clinical need to identify novel biomarkers that discriminate individuals with poor prognosis. We hypothesize that miRNAs can be used to this end because they are involved in the initiation and progression of tumors by altering the expression of their target genes. To identify a prognostic biomarker in TNBC, we analyzed the miRNA expression of a cohort composed of 185 patients diagnosed with TNBC using penalized Cox regression models. We identified a four-biomarker signature based on miR-221, miR-1305, miR-4708, and *RMDN2* expression levels that allowed for the subdivision of TNBC into high- or low-risk groups (Hazard Ratio – HR = 0.32; 95% Confidence Interval - CI = 0.11–0.91; *p* = 0.03) and are also statistically associated with survival outcome in subgroups of postmenopausal status (HR = 0.19; 95% CI = 0.04–0.90; *p*= 0.016), node negative status (HR = 0.12; 95% CI = 0.01–1.04; *p* = 0.026), and tumors larger than 2cm (HR = 0.21; 95% CI = 0.05–0.81; *p* = 0.021). This four-biomarker signature was significantly associated with TNBC as an independent prognostic factor for survival.

## Introduction

Breast cancer is the most common cancer among women, with an estimated 246,660 new cases and 40,450 deaths in 2016 in the United States (Siegel *et al.*, 2016). Based on gene expression profiling, most studies categorize breast cancer into the following four major molecular subtypes: luminal A (presence of estrogen receptor – ER^+^ and/or progesterone receptor – PR^+^, and absence of HER2 expression – HER2^-^), luminal B (ER^+^ and/or PR^+^, HER2^+^), HER2 type (ER^-^, PR^-^, HER2^+^), and triple negative (ER^-^, PR^-^, and HER2^-^) (Risbridger *et al.*, 2010).

Developments in the treatment of some types of breast cancer have increased the overall survival of patients (Vogel *et al.*, 2002; Ariazi *et al.*, 2006; Dawood *et al.*, 2009). For example, tamoxifen, which is an antagonist of the estrogen receptor, and trastuzumab, which is a monoclonal antibody that acts on the HER2 receptor, have improved the survival outcome of luminal and HER2 breast cancer subtypes, respectively. However, predictive molecular biomarkers and targeted therapies are still lacking for the treatment of triple negative breast cancer (TNBC) (Gonzalez-Angulo *et al.*, 2007; Blows *et al.*, 2010; Iwase *et al.*, 2010; The Cancer Genome Atlas Network, 2012). This may be the reason for the low improvement of survival rates of TNBC patients in the last years (Gonzalez-Angulo *et al.*, 2007). TNBC is a very heterogeneous disease, which comprises between 12% and 24% of all breast cancers and is associated with early recurrence of disease, a more aggressive phenotype, and a worse clinical prognosis than luminal and HER2 types (Gasparini *et al.*, 2014). Currently, the only option of treatment for patients diagnosed with TNBC is chemotherapy, which has limited benefits to a subgroup of patients. Thus, a molecular stratification of TNBC based on molecular markers is essential to identify novel targets of drugs.

We focused our research on microRNAs (miRNAs) because they are widely known to be critical components in cancer progression and drug resistance. miRNAs are small (19 – 25 nucleotides) single-stranded non-coding RNA molecules that regulate protein-coding genes through sequence-specific binding to 3’ untranslated regions (UTRs) of messenger RNAs (mRNA) (Zeng *et al.*, 2003; Bartel, 2004). As a consequence, they alter the abundance of gene expression, usually reducing gene expression, but a few reports have suggested that they may also induce gene expression (Vasudevan *et al.*, 2007; Place *et al.*, 2008).

Compelling evidence has shown that aberrantly expressed miRNAs are involved in the initiation and progression of human cancers and have considerable potential for use as biomarkers for the detection, diagnosis, classification, and treatment of cancer. Different tissue types present unique expression levels of each miRNA and thereby present unique miRNA “signatures.” Similarly, each tumor type presents a unique miRNA signature, which can be used to identify the tissue of origin of metastatic tumors and to discriminate between distinct cancer subtypes (Lu *et al.*, 2005). Thus, the sub-classification of TNBC using miRNAs may identify new screening methods, prognostic factors, and potential targets for personalized medicine (Cascione *et al.*, 2013).

In an attempt to identify molecular prognostic markers specific to TNBC, we analyzed a large TNBC cohort composed of 185 patients and approximately 850 miRNAs (Dvinge *et al.*, 2013). By computational analysis, we identified a four-biomarker signature that is statistically associated with patients’ outcomes in both training and validation TNBC sets.

## Materials and Methods

### MicroRNA expression data

A collection of clinically annotated and previously pre-processed public miRNA/mRNA gene expression data (Agilent) composed of approximately 850 miRNAs (including putative miRNAs) and 185 subjects diagnosed with TNBC were downloaded from the European Genome-phenome Archive webpage. This dataset (ID EGAS00000000122) was derived from fresh-frozen cancer specimens from tumor banks in the United Kingdom and Canada. The treatments were homogeneous for clinically relevant groupings. The selection criterion for the TNBC patients was based on the presence or absence of *ER*, *PR*, and *HER2* empirical gene expression distributions. To define the presence or absence of specific gene expression, Curtis *et al.* (2012) clustered the individuals into two groups by using a clustering expectation-maximization algorithm (Gaussian mixture model). Individuals belonging to the cluster with lower gene expression were set as with absent gene expression and otherwise as present. They carried out this procedure for each one of the three genes (*ER*, *PR*, and *HER2*). We selected all individuals with simultaneously absent *ER*, *PR*, and *HER2* gene expression. Basic information regarding this 185 patients data set is as follows (mean ± standard deviation or number of individuals): age at diagnosis (54.99 ± 14.37 years old), tumor size (2.72 ± 18.25 cm), presence (87), absence (95), and unknown (3) of lymph nodes, grades 1 (1), 2 (20), 3 (159), and unknown (5), subjects in premenopausal (70), postmenopausal (114), and unknown (1), histological type (infiltrating ductal carcinoma (171), infiltrating lobular carcinoma (7), ductal carcinoma in situ (2), invasive tumor (3), and benign (2)).

The 185 patients were randomized and split into training (n = 120) and validation (n = 65) sets. The clinical and pathological characteristics of both training and validation sets are summarized in Table 1.

**Table 1:** Clinical and pathological characteristics of patients and their tumors in both the training and validation sets. The data are numbers (%) unless otherwise stated. SD: standard deviation.

Variables	Status	Training set (n=120)	Validation set (n=65)
Age at diagnosis (years)	Mean (SD)	55.93 (14.47)	53.25 (14.13)
	≤40	22 (18.3)	13 (20.0)
	41-55	33 (27.5)	21 (32.3)
	56-70	47 (39.2)	24 (36.9)
	> 70	18 (15.0)	7 (10.8)
Tumor size (cm)	Mean (SD)	2.76 (1.96)	2.64 (1.49)
	≤2cm	50 (41.7)	27 (41.5)
	> 2cm	69 (57.5)	36 (55.4)
	Unknown	1 (0.8)	2 (3.1)
Lymph nodes	Negative	59 (49.2)	28 (43.1)
	Positive	60 (50.0)	35 (53.8)
	Unknown	1 (0.8)	2 (3.1)
			
Grade	1	1 (0.8)	0 (0)
	2	14 (11.7)	6 (9.2)
	3	101 (84.2)	58 (89.2)
	Unknown	4 (3.3)	1 (1.6)
Menopausal status	Premenopausal	45 (37.5)	25 (38.5)
	Postmenopausal	75 (62.5)	39 (60.0)
	Unknown	0 (0)	1 (1.5)
			
Dead	Yes	54 (46.7)	27 (41.5)
	No	64 (53.3)	39 (58.5)

### Statistical analysis

**Figure 1:**
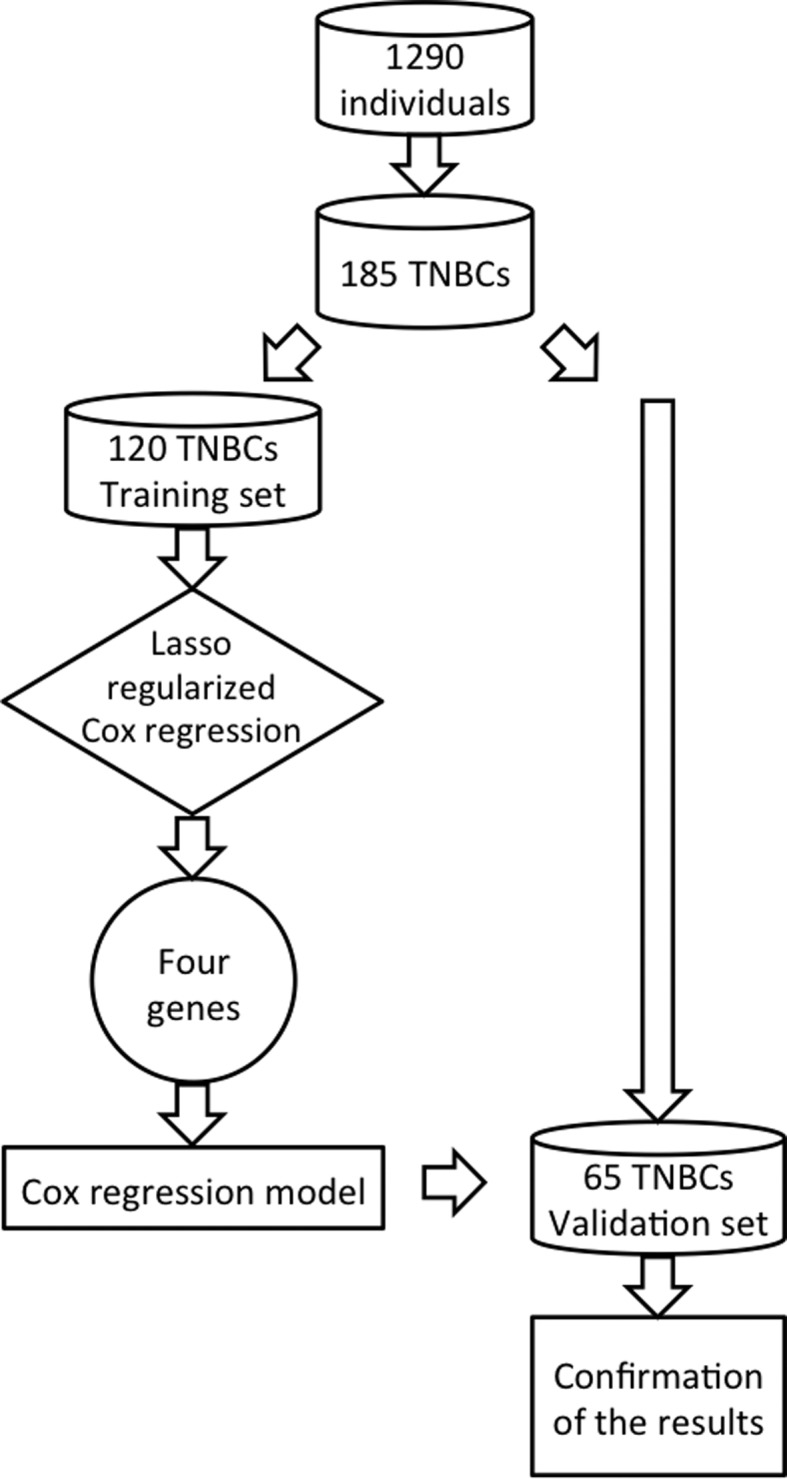
Scheme for the data analysis. We selected 185 individuals with an absent expression of ER, PR, and HER2 from a breast cancer data set composed of 1,290 individuals. This subset of 185 TNBCs was split into training (120 individuals) and validation (65 individuals) sets. To select biomarkers associated with overall TNBC survival, we used the Lasso regularized Cox regression model on the training set. Four genes were selected by the method. Then, to better estimate the parameters of the model, we used the standard Cox regression model. Finally, we confirmed the results obtained in the training set by applying the four-biomarkers in the validation set.

Because the number of miRNAs (approximately 850) is higher than the number of observations in the training set (n=120), standard Cox proportional hazard regression is not applicable. Thus, we first used the lasso regularized Cox regression for feature selection (Friedman *et al.*, 2010) to select the miRNAs that are mostly associated with overall survival time. Then, to accurately estimate the weights of each feature selected by the penalized Cox regression model (coefficients estimated by lasso regularized Cox regression are known to be biased due to l_1_ penalization), we applied the standard Cox regression. Figure 1 summarizes the analysis in a flowchart. To minimize the influence of clinical variables, such as age at diagnosis, tumor grade, size, presence/absence of nodes, and menopausal status, we included them as covariates in the standard Cox regression model.

The risk score for each patient was calculated by multiplying the expression level of each miRNA by its corresponding coefficient obtained by the Cox regression in the training set and summing them. Patients were thus dichotomized into the groups at good or poor prognosis (low or high risk) using the median cutoff of the risk score as the threshold value.

Kaplan-Meier survival curves and log-rank tests were constructed to evaluate the differences in the overall survival time of predicted good and poor prognosis groups by the miRNA signature in both the training and validation sets. Univariate and multivariate analyses with the Cox proportional hazard model were used to assess the prognostic value of the miRNA signature with and without adjusting for individual clinical prognostic variables (age at diagnosis, tumor grade, tumor size, presence/absence of nodes, and menopausal status), respectively.

To investigate the differential expression of miRNAs between good and poor prognosis, we performed a Wilcoxon signed-rank test, which is more robust to outliers than the t-test.

To identify the correlation between miRNAs and their respective target gene expressions, we used the Spearman correlation.

All computations were performed in the R statistical environment (R Core Team, 2014). Both lasso regularized and standard Cox regressions were performed by using R packages, namely *glmnet* (with parameters alpha = 1 and family = “cox”) and *survival*, respectively.

### Data access

The data that support the findings of this study are available from https://www.ebi.ac.uk/ega/, but restrictions apply to the availability of these data, as these were used under license for the current study. Thus, these data are not publicly available. However, these data are available from the authors upon reasonable request and with permission of the Data Access Committee.

## Results

### Development of a miRNA prognostic signature for TNBC patients

The entire set of approximately 850 miRNAs was analyzed to develop a prognostic signature in the training set. One subject diagnosed with grade I was removed from our analysis due to its low quantity.

**Figure 2:**
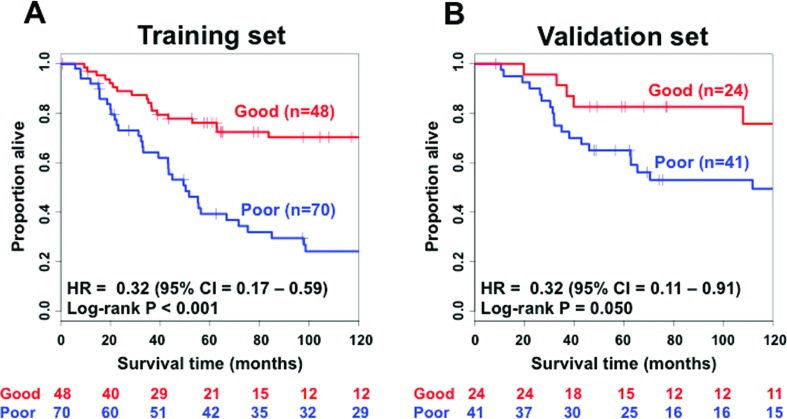
Kaplan-Meier analysis showing that the four-biomarker signature is associated with survival in triple negative breast cancer. (A) Kaplan-Meier curve of the four-biomarker signature in the training set. (B) Kaplan-Meier curve of the four-biomarker signature in the validation set. CI = confidence interval; HR = hazard ratio. HR and 95% CI were estimated by multivariate Cox regression with age at diagnosis, grade, the presence of nodes, and menopausal status included as covariates. The *p*-value was obtained by the log-rank test of the Kaplan-Meier curve.

By performing the lasso regularized Cox regression analysis, we identified four miRNAs (two of them were putative) as being associated with overall survival in TNBC, namely miR-221 (probe ID: A_25_P00010690), miR-1305 (probe ID: A_25_P00015133), miR-11624 (probe ID: CRINCR2000004678, putative miRNA), and miR-10055 (probe ID: CRINCR2000004642, putative miRNA). We contacted the authors of the original study (Dvinge *et al.*, 2013), who provided us with the probe sequences of the two putative miRNAs. Then, we re-annotated them by mapping the probe sequences to the human genome version hg38 by BLAT (Kent, 2002). The putative microRNAs miR-11624 and miR-10055 mapped to (with 100% alignment) the previously annotated miR-4708 and an exon of gene *RMDN2* (regulator of microtubule dynamics 2), respectively. Therefore, our prognostic signature is composed of three microRNAs and one gene that codes for a protein, specifically miR-221, miR-1305, miR-4708, and *RMDN2*. Next, a prognostic model composed of these three miRNAs and the *RMDN2* gene was constructed by using the standard Cox proportional hazard model. Associated coefficients and hazard ratios obtained in both univariate and multivariate (including clinical covariates) Cox proportional hazard models are described in Table 2. The Kaplan-Meier curve discriminating subjects classified with good or poor prognosis (log-rank test *p* < 0.001) in the training set is shown in Figure 2A. The four-biomarker signature (Table 2) is indeed statistically associated with overall survival time in TNBC even after inclusion of clinicopathological covariates (univariate: HR = 0.29, 95% CI: 0.16 – 0.52, *p* < 0.001; multivariate: HR = 0.32, 95% CI: 0.17 – 0.59, *p* < 0.001).

**Table 2:** Univariate and multivariate analyses of the four-biomarker signature. Univariate and multivariate analyses were performed based on the Cox regression model. CI: confidence interval.

	Training set			
	Univariate analysis		Multivariate analysis	
Variables	Hazard ratio (95%CI)	*p*-value	Hazard ratio (95%CI)	*p*-value
Age at diagnosis	1.12 (0.85-1.47)	0.407	1.12 (0.70-1.80)	0.642
Tumor grade (II/III)	3.00 (0.94-9.64)	0.065	2.59 (0.79-8.50)	0.116
Tumor size	1.27 (1.07-1.50)	0.006	1.06 (0.88-1.27)	0.540
Presence of nodes	1.44 (1.21-1.71)	<0.001	1.35 (1.11-1.64)	0.002
Menopausal status (pre/pos)	1.04 (0.60-1.83)	0.883	1.30 (0.46-3.66)	0.623
Four-biomarker signature	0.29 (0.16-0.52)	<0.001	0.32 (0.17-0.59)	<0.001

	Validation set			
	Univariate analysis		Multivariate analysis	
Variables	Hazard ratio (95%CI)	*p*-value	Hazard ratio (95%CI)	*p-*value
Age at diagnosis	1.27 (0.88-1.85)	0.207	1.38 (0.74-2.65)	0.306
Tumor grade (II/III)	2.73 (0.37-20.22)	0.327	2.08 (0.27-16.09)	0.484
Tumor size	1.42 (0.98-2.05)	0.065	1.35 (0.92-1.97)	0.124
Presence of nodes	1.51 (0.98-2.30)	0.059	1.62 (0.96-2.72)	0.071
Menopausal status (pre/pos)	0.68 (0.52-2.73)	0.675	0.67 (0.17-2.73)	0.579
Four-biomarker signature	0.39 (0.14-1.04)	0.059	0.32 (0.11-0.91)	0.033

**Figure 3:**
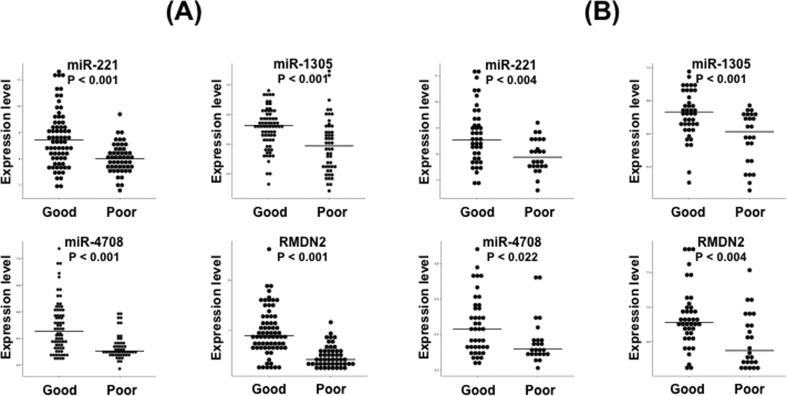
Expression of miR-221, miR-1305, miR-4708, and *RMDN2* between good and poor prognosis in the (A) training and (B) validation sets. Horizontal bars represent the median. All genes are statistically overexpressed in patients with a good prognosis at a *p*-value threshold of 0.05.

Wilcoxon signed-rank tests showed that all four biomarkers (miR-221: *p* < 0.001, miR-1305: *p* < 0.001, miR-4708: *p* < 0.001, and *RMDN2*: *p* < 0.001) are significantly overexpressed in patients classified with good prognosis in the training set (Figure 3A).

### Validation of the four-biomarker signature in a test set

**Figure 4:**
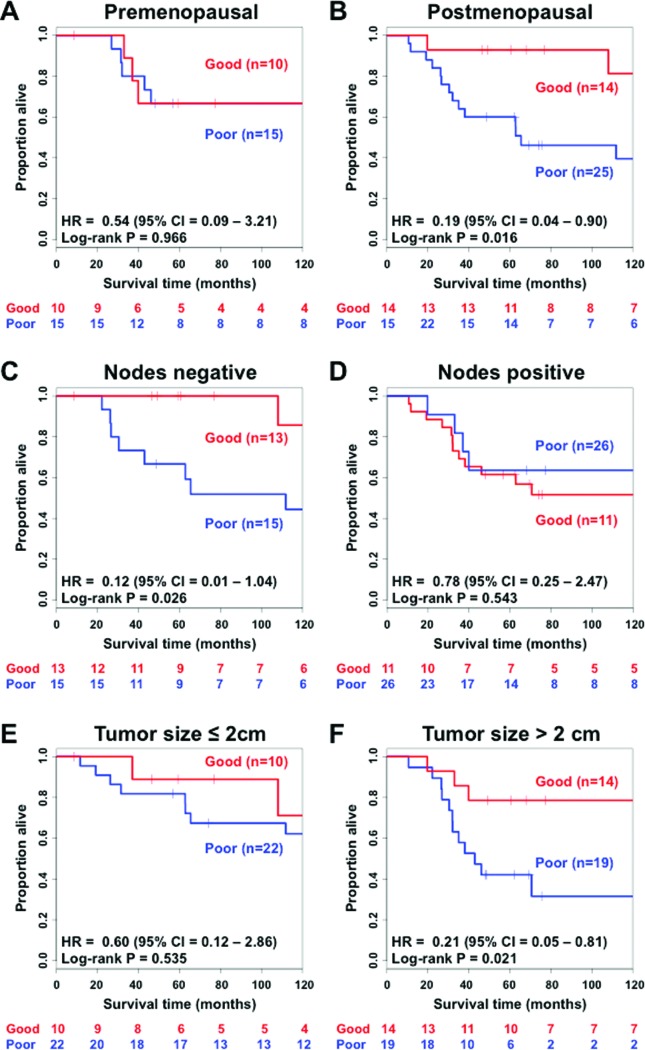
Kaplan-Meier analysis of overall survival in subgroups of triple-negative breast cancer patients in the validation set. (A) Premenopausal patients; (B) postmenopausal patients; (C) patients without nodes identified; (D) patients with nodes; (E) patients with a tumor size of 2 cm or less; (F) patients with tumor size greater than 2 cm. Hazard ratios and confidence intervals were obtained by multivariate Cox regression with age at diagnosis, tumor grade, tumor size, and presence of nodes status as covariates. *P*-values were obtained by log-rank tests for the Kaplan-Meier curves. HR = hazard ratio; CI = confidence interval.

The Cox proportional hazard model obtained in the training set was applied to 65 subjects of the testing set and validated. The Kaplan-Meier curves are shown in Figure 2B (log-rank test *p* = 0.050). Hazard ratios and respective *p*-values of both univariate and multivariate Cox proportional hazard models are shown in Table 2. The four-biomarker signature is an independent prognostic factor (univariate: HR = 0.39, 95% CI: 0.14 – 1.04, *p* < 0.059; multivariate: HR = 0.39, 95% CI: 0.14 – 1.04, *p* = 0.033) after inclusion of the covariates. Subgroups of patients with postmenopausal status (Figure 4B - HR=0.19; 95% CI = 0.04 – 0.90; *p* = 0.016), node negative status (Figure 4C – HR = 0.12; 95% CI = 0.01 – 1.04; *p* = 0.026), and a tumor size greater than the median, i.e., 2 cm (Figure 4F – HR = 0.21; 95% CI = 0.05 – 0.81; *p* = 0.021) also showed that the four-biomarker signature was statistically associated with overall survival. Subgroups comprising premenopausal status (Figure 4A – HR = 0.54; 95% CI = 0.09 – 3.21; *p* = 0.966), node positive status (Figure 4D – HR = 0.78; 95% CI = 0.25 – 2.47; *p* = 0.543), and a tumor size smaller than 2 cm (Figure 4E – HR = 0.60; 95% CI = 0.12 – 2.86; *p* = 0.535) do not present statistical evidence of associations between the four-biomarker signature and survival outcome at a *p*-value threshold of 0.05.

Wilcoxon signed-rank tests showed that all four biomarkers (miR-221: *p* = 0.004, miR-1305: *p* = 0.001, miR-4708: *p* = 0.022, and *RMDN2*: *p* = 0.004) are also significantly overexpressed in patients classified with good prognosis in the validation set, confirming the results obtained in the training set (Figure 3B).

To confirm that indeed all four biomarkers are essential for classification into good or poor prognoses, we performed the following experiment: four competing three-biomarker signatures were designed by deleting one of the four genes from the set. Then, we repeated the survival analysis for each of these three-biomarker signatures. The results showed that none of the three-gene signatures was statistically associated with overall survival in the testing data set, confirming that all four biomarkers are, in fact, necessary (Figure S1).

**Figure 5:**
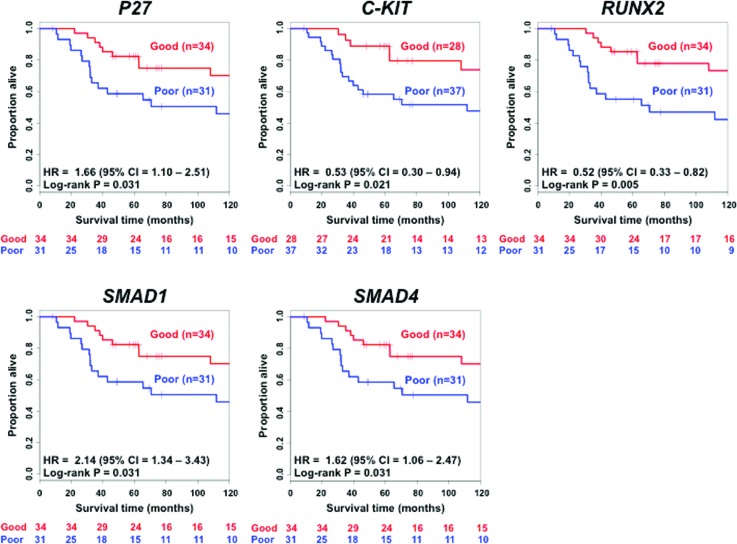
Kaplan-Meier analysis showing that the five target genes are associated with survival in triple negative breast cancer. The *p*-value is obtained by the log-rank test of the Kaplan-Meier curve.

**Figure 6:**
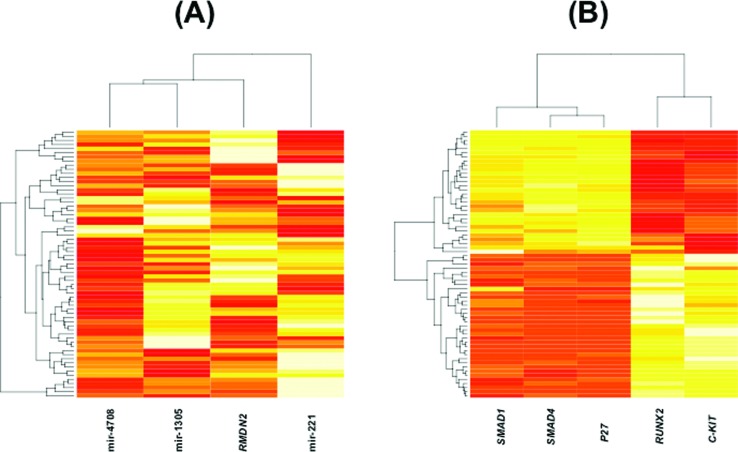
Gene expression heatmaps. (A) For the four prognostic biomarkers and (B) the five genes targets of the three miRNAs. In panel (A), the four biomarkers are not correlated, while in panel (B) there are two sets of genes: one composed of genes *SMAD1*, *SMAD4*, and *p27*, and the second set composed of *RUNX2* and *c-kit*. Genes within the sets are positively highly correlated, while between sets, they are negatively highly correlated.

Next, we analyzed the gene expression patterns between the identified miRNAs and their known target genes. We identified a statistically significant negative association between miR-221 and *p27* (Spearman correlation = -0.34; *p* < 0.005), but not between miR-221 and *c-kit* (Spearman correlation = 0.18; *p*-value = 0.158), miR-1305 and *RUNX2* (Spearman correlation = -0.14; *p*-value = 0.282), miR-4708 and *SMAD1* (Spearman correlation = -0.14; *p*-value = 0.250), and miR-4708 and *SMAD4* (Spearman correlation = 0.18; *p*-value = 0.158). We also constructed Kaplan-Meier curves for the five target genes. Figure 5 shows that all five target genes are also associated with TNBC overall survival. Figure 6A shows the heatmaps for both the four biomarkers and Figure 6B for their target genes (*p27*, *c-kit*, *RUNX2*, *SMAD1*, and *SMAD4*) in the 65 individuals composing the validation set. To verify whether the categorizations as good or poor prognosis by the four-biomarker set are similar to the ones obtained by each one of the five target genes, we calculated the Spearman correlation coefficient. As results, we obtained the following: *p27* (Spearman correlation = 1; *p*-value < 0.001), *c-kit* (Spearman correlation = 0.80; *p*-value < 0.001), *RUNX2* (Spearman correlation = 0.81; *p*-value < 0.001), *SMAD1* (Spearman correlation = 1; *p*-value < 0.001), and *SMAD4* (Spearman correlation = 1; *p*-value < 0.001). In other words, the classification of TNBC patients into low or high risk obtained by the four-biomarker is very similar to the classification obtained by each target gene independently.

## Discussion

By analyzing a large TNBC cohort, we identified a four-biomarker signature composed of miR-221, miR-1305, miR-4708, and *RMDN2* in a training set and then confirmed their association with prognosis in a validation set.

miR-221 has been associated with several cancers, such as thyroid papillary (Visone *et al.*, 2007), liver (Pineau *et al.*, 2010), and prostate cancers (Fornari *et al.*, 2008). miR-221 directly interacts with cyclin-dependent kinase inhibitors CDKN1B/*p27* and CDKN1C/*p57* by binding to their mRNA 3’UTR (le Sage *et al.*, 2007; Visone *et al.*, 2007; Fornari *et al.*, 2008; Pineau *et al.*, 2010). Galardi *et al.* (2007) and Visone *et al.* (2007) showed that the expression levels of miR-221 and *p27* are negatively correlated, which was also confirmed in our TNBC dataset. Thus, the down-regulation of miR-221 in the group classified with poor prognosis might lead to increased *p27* expression.

P27 is a negative regulator of cell cycle progression, and the loss of this protein is often observed in several cancers (le Sage *et al.*, 2007; Visone *et al.*, 2007; Fornari *et al.*, 2008; Pineau *et al.*, 2010). The increased *p27* expression through miR-221 down-regulation in patients classified with poor prognosis might induce cell cycle arrest, resulting in resistance to chemotherapy (chemotherapy is useful in highly proliferative cancer cells, but it is not effective in quiescent cells or slow-cycling). Cancer stem cells, which are responsible for cancer metastasis and recurrence, are generally quiescent or slow-cycling and resistant to conventional chemo- and radio-therapies (Yoshida and Saya, 2016). Zou *et al.* (2011) reported that p27 and p57 control hematopoietic stem cells dormancy, while Besson *et al.* (2007) showed an association between p27 and bronchioalveolar stem cell expansion. Moreover, it is also known that p27 prevents the activation of RhoA (Besson *et al.*, 2004; Wang and Lee, 2014) and that the inactivation of the RhoA-ROCK pathway enhances cancer stem cell propagation (Ohata *et al.*, 2012; Tilson *et al.*, 2015).

Another possible pathway influenced by miR-221 and also related to tumorigenesis is angiogenesis. Endothelial cells transfected *in vitro* with miR-221 have inhibited tube formation, migration, and wound healing properties (Poliseno *et al.*, 2006). The underlying mechanism involves the down-regulation of the protein c-kit, a receptor for stem cell factor, without affecting the mRNA level, suggesting a post-transcriptional regulation (Poliseno *et al.*, 2006). Indeed, even in our TNBC data set, we did not identify the correlation between miR-221 and *c-kit* expression. In hematopoietic progenitor cells, miR-221 reduces *c-kit* expression and thus reduces cell proliferation (Felli *et al.*, 2005). Overexpression of miR-221 indirectly reduces the expression of endothelial nitric oxide synthase (NOS) in Dicer siRNA-transfected cells (Suarez *et al.*, 2007). Nitric oxide is a crucial regulator for endothelial cell growth, migration, vascular remodeling, and angiogenesis (Zeiher, 1996). Recently, it was demonstrated that endothelial NOS plays a crucial role in the mobilization and functional activity of stem and progenitor cells (Aicher *et al.*, 2003; Iwakura *et al.*, 2003; Landmesser *et al.*, 2004).

Changes in the miR-1305 expression are associated with tumorigenesis in several tissues (Niyazi *et al.*, 2011; Shah *et al.*, 2013; Tormo *et al.*, 2015). *RUNX2* is a direct target of miR-1305 (Chen and Liu, 2017), and its up-regulation is associated with a variety of cancer tissues (Brubaker *et al.*, 2003; Kayed *et al.*, 2007; Endo *et al.*, 2008). More specifically, in breast cancer, Runx2 has been demonstrated to promote bone metastasis (Javed *et al.*, 2005; Li *et al.*, 2015). The inhibition of miR-1305 expression in TNBC patients may lead to an increase in Runx2 expression via a post-transcriptional mechanism (because we did not identify the correlation between them) that promotes breast cancer aggressiveness.

Overexpression of miR-4708 directly inhibits *SMAD1* and *SMAD4* gene expression (Kato *et al.*, 2014). The SMAD family is a group of transcription factors coding regulatory genes that mediate the TGF-β pathway, which controls the cell cycle and growth (Massagué, 2000; Derynck *et al.*, 2001). The TGF-β pathway can inhibit cell proliferation by activating receptor-regulator SMADs (SMAD1, 2, 3, 5, and 8), which activate SMAD4. These molecules are then transferred to the nucleus to control gene expression (Shi and Massague, 2003).

Consistent with the tumor inhibitory role of the TGF-β pathway, the association between deregulation of *SMAD*s and tumor proliferation has been found in different cell types. Increased BMPR-IB (a protein of TGF-β pathway) expression leads to a deregulated SMAD1 activity, which was already reported to be associated with an increase in breast cancer progression (Helms *et al.*, 2005). Inactivating mutations in *SMAD4* have been found in different tumor types (Hahn *et al.*, 1996; Schutte *et al.*, 1996; Miyaki *et al.*, 1999), including breast cancers (Levy and Hill, 2005; Deckers *et al.*, 2006). Moreover, silencing or knockout of *SMAD4* promotes cell proliferation by abrogating the TGF-β pathway (Levy and Hill, 2005; Deckers *et al.*, 2006).

Aside from the inhibitory characteristic of the TGF-β pathway on cell growth, this pathway is also central to another contradictory role, metastasis (Derynck *et al.*, 2001). Evidence suggests that the down-regulation of *SMAD4* reduces metastasis in breast cancer cells (Deckers *et al.*, 2006).

Thus, these findings suggest the following hypothesis: down-regulation of miR-4708 increases *SMAD* expression, which, in turn, activates the TGF-β pathway. TGF-β pathway activation, then, could lead to increased metastatic behavior. Since we did not identify the correlation between mir-4708 and *SMAD1* neither between mir-4708 and *SMAD4*, we believe they may be regulated by a post-transcriptional mechanism.

Finally, the *RMDN2* gene is known to code proteins that bind and regulate microtubule growth. Its mutants are associated with defective chromosome segregation (Oishi *et al.*, 2007; Law *et al.*, 2015). During anaphase, when the sister chromatids usually move in opposite directions, in *RMDN2* mutants, chromatids do not segregate; they become stretched and cut at the end of cell division (Oishi *et al.*, 2007). A genome-wide association analysis suggested a potential relationship between the *RMDN2* 3’UTR and increased susceptibility to malignant melanoma (Law *et al.*, 2015).

In summary, all four biomarkers are associated with cell cycle control and growth, pathways that are intimately related to cancer development and progression. The presented scenario supports the hypothesis that the down-regulation of these four biomarkers may be leading to more aggressive and faster-growing tumors in patients classified with a poor prognosis.

By analyzing the five genes that are targets of the three miRNAs (Figure 5), we noticed that the Kaplan-Meier curves are very similar. This can be explained by the fact that these five genes are highly correlated. Notice that in the heatmap of Figure 6B, there are two sets of genes: one composed of *SMAD1*, *SMAD4*, and *p27*, and the second one composed of *RUNX2* and *c-kit*. Within each set, the genes are positively correlated (*p* < 0.05 for all pairwise Spearman correlations within the set), while between sets, they are negatively correlated (*p* < 0.05 for all pairwise Spearman correlations between sets). No correlation was observed among the four biomarkers (*p* > 0.05 for all pairwise Spearman correlations) (Figure 6A). Interestingly, the classifications obtained by the four-biomarker set and each one of the five target genes are very similar (high correlation). This means that, although there is no correlation between the miRNA and expression of its respective target gene, the cooperative indirect regulation of the four biomarkers on the target genes might affect the TNBC malignancy (notice that in Figure S1 all four biomarkers were necessary for a proper classification).

Although these results indicate that the four-biomarker signature described here is statistically associated with TNBC survival outcome, it would be worthwhile to validate them in other independent cohorts. However, only limited comparison with published data is possible due to at least two reasons: (i) the lack of large miRNA TNBC datasets and (ii*)* the absence of these four biomarkers in other studies. For example, Lowery *et al.* (2009) and Mattie *et al.* (2006) analyzed miRNAs in small sets composed of 29 and 20 breast cancer tumors, respectively. Alternatively, Farazi *et al.* (2011), Gasparini *et al.* (2014), and Raju *et al.* (2014) provided large sets composed of 185, 219, and 587 patients of diverse types of breast cancer, respectively, but none of their datasets have the four biomarkers that we identified here.

**Figure 7:**
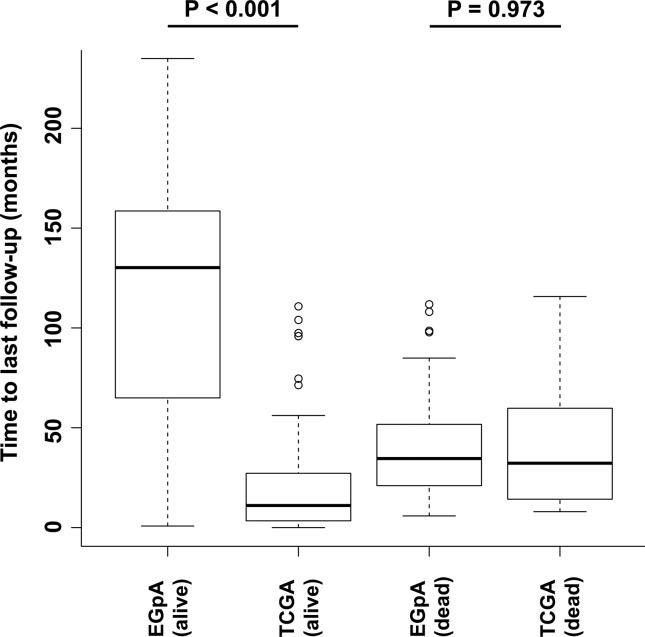
Time lengths for the last follow-ups. There is no statistical difference in terms of time to death between individuals that died in the EGpA (average of 39.44 months) and TCGA (average of 45.69 months) data sets (*p*=0.973, Wilcoxon rank test). However, when we analyzed the time length for the last follow-up of people who did not die, we noticed that EGpA has a much longer follow-up (average of 118.55 months) than TCGA (average of 21.12 months) (*p*-value < 0.001, Wilcoxon rank test).

We also tried validation by using the TCGA data set. We downloaded all the miRNA/mRNA data of 1,099 individuals diagnosed with breast cancer from the TCGA webpage. Among them, 113 were diagnosed with TNBC. We applied our Cox proportional hazard model obtained in the training set to these 113 subjects. The Kaplan-Meier curves presented a log-rank test *p*-value equal to 0.72. In other words, it was not possible to confirm our results in the TCGA data set. We have at least two possible explanations for this negative result. The first one is the low number of deaths in the TCGA data set (only 13 individuals out of 113, i.e., ~11.5%). By considering a low number of deaths, it is challenging to identify differences between the Kaplan-Meier curves, even if they exist. For comparison, notice that in the data set we used for our analyses (European Genome-phenome Archive - EGpA), the proportions of deaths are 46.7% and 53.3% in the training and validation data sets, respectively (see Table 1). The second explanation is based on the short follow-up length of the TCGA data set. In Figure 7, we show the boxplots regarding the follow-up lengths for people considered alive and dead in both EGpA and TCGA data sets. Notice that there is no statistical difference in terms of time to death between individuals that died in the EGpA (average of 39.44 months) and TCGA (average of 45.69 months) data sets (*p* = 0.973, Wilcoxon rank test). However, when we analyzed the time lengths for the last follow-up of people who survived (or did not die yet), we notice that EGpA has a much longer follow-up (average of 118.55 months) than TCGA (average of 21.12 months) (*p*-value < 0.001, Wilcoxon rank test). In other words, to better characterize the outcomes of individuals from the TCGA data set, we believe that a more extended period of follow-up is necessary.

The four-biomarker signature is statistically associated with the overall survival of TNBC patients. These results may aid in the development of better methods to predict prognosis or choose therapies for the management of TNBC patients.

## Supplementary material

The following online material is available for this article:

Figure S1 Kaplan-Meier curves for three-biomarker signatures.
