# Supplementary material for: Large miRNA survival analysis reveals a prognostic four-biomarker signature for triple negative breast cancer
Source: Genet Mol Biol. 2020 Mar 2;43(1):e20180269. doi: 10.1590/1678-4685-GMB-2018-0269 (PMC7198019; doi:10.1590/1678-4685-GMB-2018-0269)
Supplement: Supplementary file 1 [file 1415-4757-GMB-43-01-e20180269-s001.pdf]

Supplementary Material to "**Large miRNA survival analysis reveals a prognostic four-biomarker signature for triple negative breast câncer**"

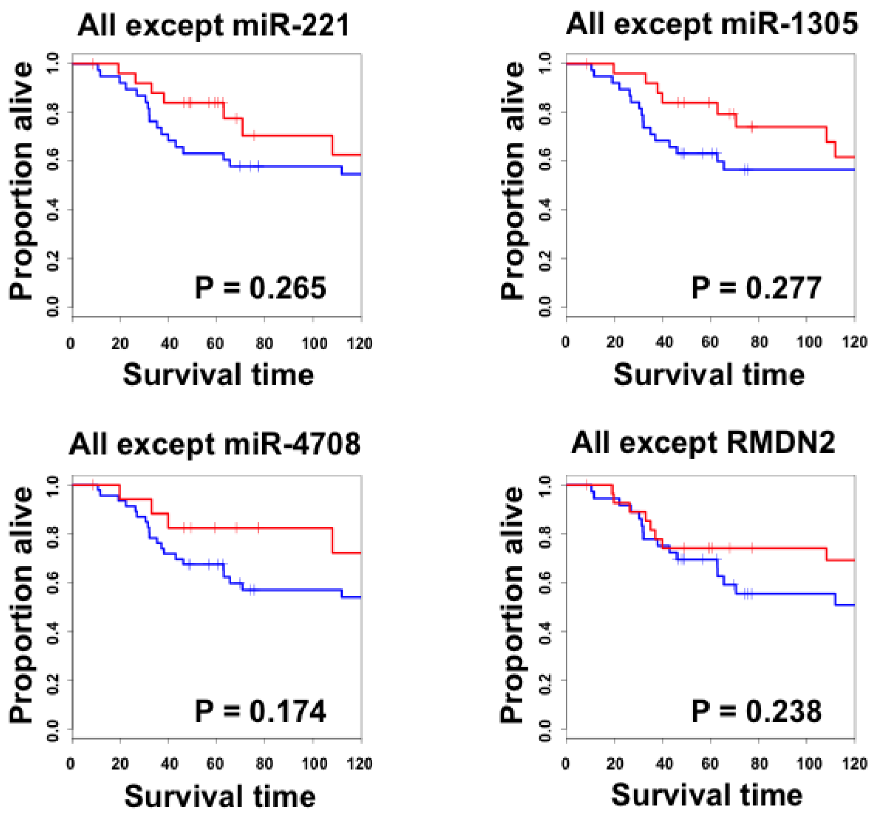

Figure S1 - Kaplan-Meier curves for the following experiment: four competing three-biomarker signatures were designed by deleting one of the four genes from the set. Then, we repeated the survival analysis for each of these three-biomarker signatures. The results showed that none of the three-gene signatures was statistically associated with overall survival in the testing data set, confirming that all four biomarkers are indeed necessary.
